# Processing of DNA double strand breaks by alternative non-homologous end-joining in hyperacetylated chromatin

**DOI:** 10.1186/2041-9414-3-4

**Published:** 2012-08-22

**Authors:** Vasilissa Manova, Satyendra K Singh, George Iliakis

**Affiliations:** 1 Institute of Medical Radiation Biology, University of Duisburg-Essen Medical School, Hufelandstr. 55, 45122, Essen, Germany; 2 Department of Molecular Genetics, Institute of Plant Physiology and Genetics, Bulgarian Academy of Sciences, Sofia, Bulgaria

**Keywords:** DNA Double strand breaks (DSB), Ionizing radiation (IR), HDAC, Chromatin, Chromatin acetylation, NHEJ

## Abstract

**Background:**

Mammalian cells employ at least two subpathways of non-homologous end-joining for the repair of ionizing radiation induced DNA double strand breaks: The canonical DNA-PK-dependent form of non-homologous end-joining (D-NHEJ) and an alternative, slowly operating, error-prone backup pathway (B-NHEJ). In contrast to D-NHEJ, which operates with similar efficiency throughout the cell cycle, B-NHEJ operates more efficiently in G2-phase. Notably, B-NHEJ also shows strong and as of yet unexplained dependency on growth activity and is markedly compromised in serum-deprived cells, or in cells that enter the plateau-phase of growth. The molecular mechanisms underpinning this response remain unknown. Since chromatin structure or changes in chromatin structure are prime candidate-B-NHEJ-modulators, we study here the role of chromatin hyperacetylation, either by *HDAC2* knockdown or treatment with the HDAC inhibitor TSA, on the repair by B-NHEJ of IR-induced DSBs.

**Results:**

siRNA-mediated knockdown of *HDAC2* fails to provoke histone hyperacetylation in *Lig4*^*-/-*^ MEFs and has no detectable effect on B-NHEJ function. Treatment with TSA that inhibits multiple HDACs causes efficient, reversible chromatin hyperacetylation in *Lig4*^-/-^ MEFs, as well as in human HCT116 *Lig4*^-/-^ cells and the human glioma cell line M059K. The IR yield of DSBs in TSA-treated cells remains similar to that of untreated cells despite the expected chromatin relaxation. In addition, chromatin hyperacetylation leaves unchanged repair of DSBs by B-NHEJ in irradiated exponentially growing, or plateau-phase cells. Notably, under the experimental conditions employed here, chromatin hyperacetylation fails to detectably modulate B-NHEJ in M059K cells as well.

**Conclusions:**

In summary, the results show that chromatin acetylation or deacetylation does not affect the kinetics of alternative NHEJ in all types of cells examined both in exponentially growing and serum deprived cultures. We conclude that parameters beyond chromatin acetylation determine B-NHEJ efficiency in the plateau-phase of growth.

## Background

It is commonly believed that DSBs induced in the genome of higher eukaryotes by widely diverse endogenous and exogenous factors and processes are mainly repaired by non-homologous end-joining (NHEJ) [1-3]. The canonical and widely investigated pathway of NHEJ (D-NHEJ) starts with the binding to the generated ends of the Ku70/Ku80 complex, which then helps recruit the DNA-dependent protein kinase (DNA-PK) as well as other factors, including the nuclease Artemis and the Lig4/Xrcc4/XLF complex. End-joining occurs rapidly, with only minimal processing of the DNA ends to render them ligatable and limited polymerization [2].

When D-NHEJ fails, locally in repair proficient cells, and globally in mutants with defects in D-NHEJ components, or in cells treated with DNA-PK inhibitors, an alternative form of end joining operating as backup to D-NHEJ becomes activated (B-NHEJ) [1,4-6]. B-NHEJ utilizes Lig3 and Parp1 [7-11], but also histone H1 as a stabilizing factor [12] and BCR/Abl as a regulatory component [13,14]. Also components of the DNA end-resection apparatus such as the MRN complex and CtIP are implicated in B-NHEJ [15-20].

B-NHEJ contributes to important cellular functions. It robustly supports class-switch recombination at the Ig locus [21,22], and V(D)J recombination in B cells harboring mutant forms of Rag1 and Rag2 that release unrejoined ends for processing by pathways other than D-NHEJ [23]. B-NHEJ also supports telomere maintenance [24]. On the negative hand, B-NHEJ is directly implicated in the formation of chromosome aberrations and thus also in carcinogenesis [5,6,9,17,19].

B-NHEJ shows dependence throughout the cell cycle that is fundamentally different from that of other DSB repair pathways [4]. It is well documented that D-NHEJ operates throughout the cell cycle and homologous recombination repair (HRR) only during the S- and G2 phase of the cell cycle, where a sister chromatid becomes available. In contrast, B-NHEJ remains active throughout the cell cycle, like D-NHEJ, but shows a marked enhancement during the G2 phase like HRR [25,26]. An additional and probably more intriguing feature of B-NHEJ is the strong growth-state dependence it shows. Thus, B-NHEJ is markedly compromised in cells that enter the plateau-phase of growth [27,28]. This effect has been recently reproduced in cultures deprived of serum [29]. The reduction of B-NHEJ activity in non-cycling cells is profound and comparable to that observed for D-NHEJ between *Ku70/Ku80* or *Lig4* mutants and wild type cells. It suggests important regulatory mechanisms that remain to be elucidated. The present work is conceived as an attempt to elucidate parameters underpinning this response and focuses on chromatin conformation as a possible modulator of B-NHEJ efficiency.

Changes in chromatin conformation facilitate several DNA repair pathways [30-33] and play a central role in DNA damage signaling [34-37]. Histone H1 features as a stimulatory factor of B-NHEJ in a biochemical screen [12] and heterochromatin is thought to present a barrier that determines DSB repair pathway selection [38-40]. Yet, the role of chromatin conformation and chromatin compactness in B-NHEJ remains unknown, although it may partly underpin the marked efficiency fluctuations observed with cell cycle phase and growth state.

Histone acetylation, together with DNA methylation, plays a crucial role in chromatin dynamics [41]. Acetylation neutralizes the strong positive charge of histones and is associated with relaxed chromatin, whereas histone deacetylation is a hallmark of compacted and thus inaccessible chromatin. Histone acetylation is regulated by the concerted action of histone acetyltransferases and histone deacetylases (HDACs) that add or remove, respectively, acetyl groups from lysine residues [42,43]. There are 18 known HDACs in human cells falling into four classes. Class I is related to budding yeast Rpd3 and includes the proteins HDAC1, HDAC2, HDAC3 and HDAC8 that are ubiquitously expressed and mainly localized in the nucleus. Class II HDACs are related to yeast Hda1 and includes the proteins HDAC4-7, HDAC9 and HDAC10; they are not ubiquitously expressed and are mainly localized in the cytoplasm. Class III HDACs known as sirtuins, are related to yeast Sir2 and includes the proteins SIRT1-7 that can be nuclear or cytoplasmic. Class IV HDACs consists of only HDAC11 [44-46].

Trichostatin A (TSA) is an aliphatic, hydroxamic-acid-based compound, which exhibits strong inhibitory activity on both class I and class II HDACs. Its mode of inhibition is thought to be through chelation of the zinc ion at the catalytic site of HDAC [47], which prevents the multiprotein complex from removing the acetyl group from the lysine residues of histones. Treatment of cells with TSA provokes histone acetylation and chromatin relaxation [48], but also cell cycle arrest [49].

The levels of chromatin acetylation or changes in chromatin acetylation have widely different and possibly context-dependent effects on DNA repair [31]. In murine cells histone hypoacetylation results in defective recruitment of DNA repair factors and compromises DSB repair, while hyperacetylation mediated by treatment with HDAC inhibitors allows efficient recruitment of HRR proteins [50]. On the other hand, treatment with HDAC inhibitors suppresses D-NHEJ-factor expression and causes cell radiosensitization to killing [33,51]. Also, a delaying effect of HDAC inhibitors on both HRR and NHEJ has been observed [52].

While it is thought that nucleosome unfolding and relaxation facilitates D-NHEJ [53], chromatin compactness may also contribute to efficient NHEJ by keeping the two DNA ends of a DSB close together [54]. Thus, chromatin conformation can be either a facilitator or an impediment of DSB repair. Indeed, chromatin compactness contributes to the efficient and correct rejoining of IR-induced DSBs in centromeric DNA [55]. On the other hand, access of D-NHEJ factors to DSBs in transcriptionally active genomic regions enhances repair [56]. Recent work also shows that DSB repair within heterochromatic regions is facilitated by modulations in chromatin compactness, suggesting that transient conformational alterations are integrated in DSB repair pathways more than previously thought [40,57].

How the chromatin state or changes in chromatin conformation affect B-NHEJ remains unknown, although effects such as the marked reduction in B-NHEJ efficiency in non-cycling cells point to chromatin conformation as a candidate parameter. To begin addressing the role of chromatin on B-NHEJ efficiency we examine here the effect of chromatin hyperacetylation induced either by treatment with TSA or via *HDAC* knockdown on B-NHEJ function.

## Results

### Effect on B-NHEJ of *HDAC2* knockdown

We first inquired whether depletion of individual HDACs modulates the efficiency of B-NHEJ either in actively growing, or serum-deprived D-NHEJ deficient cells [29]. For this purpose *Lig4*^-/-^ MEFs were employed because their deficiency in Lig4 compromises D-NHEJ and allows B-NHEJ to dominate repair of IR induced DSBs. Among HDACs, we selected for knockdown the transcriptional co-repressor HDAC2, as its depletion correlates with chromatin decondensation and increased DNA accessibility [33].

For efficient silencing of *HDAC2* a mixture of four siRNAs was used. Additional file 1A demonstrates over 80% knockdown of *HDAC2*, 24-48 h after transfection. *HDAC2* knockdown was also confirmed by real-time RT-PCR ( Additional file 1B). FACS data obtained 24-72 h after transfection show that *HDAC2* knockdown has no effect on cell cycle distribution. The accumulation of cells in G1 after 72 h reflects the progression of cells into a plateau-phase ( Additional file 1C). Based on this data, experiments on B-NHEJ function were carried out 28-36 h after siRNA transfection.

**Figure 1:**
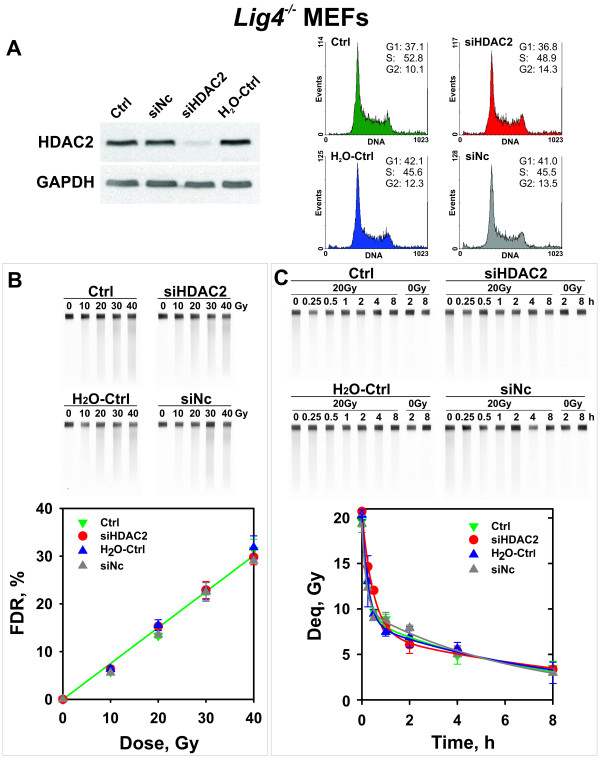
**Effect of *HDAC2 *silencing on B-NHEJ in exponentially growing *Lig4***^***-/- ***^**MEFs.(A)** Western blot showing HDAC2 levels 30 h after transfection with siRNA targeting *HDAC2* together with the corresponding controls: siNc – negative control siRNA with no target in MEF genome; H_2_O-Ctrl – water control; Ctrl - non-treated control. GAPDH represents a loading control. Right panel: Cell cycle distribution of the populations employed in the repair experiment shown in the following panels. **(B)** Yields of DSBs measured by PFGE in cells pre-treated as indicated. The upper panel shows typical gels while the lower panel the average and standard error of FDR calculated from three independent experiments, 2-3 determinations per experiment. **(C)** Kinetics of rejoining of DSBs in *Lig4*^*-/-*^ MEFs exposed to 20 Gy X-rays. The mean and standard errors of Deq calculated from 4-7 determinations in three independent experiments are shown. Other details are as in panel B.

The effect of *HDAC2* knockdown on DSB induction and repair in *Lig4*^-/-^ MEFs is shown in Figure 1. Figure 1A shows efficient knockdown of *HDAC2* 30 h after transfection without detectable effects on cell cycle distribution. At this point changes in the induction of DSBs cannot be detected (Figure 1B) and the fraction of DNA released from the well into the lane (FDR, see “Methods” for definitions) is similar under all conditions tested. Also the kinetics of DSB repair plotted as equivalent dose versus time (Deq, see “Methods” for definitions) (Figure 1C) remains unchanged after *HDAC2* knockdown. We conclude HDAC2 has no detectable essential contribution to B-NHEJ.

**Figure 2:**
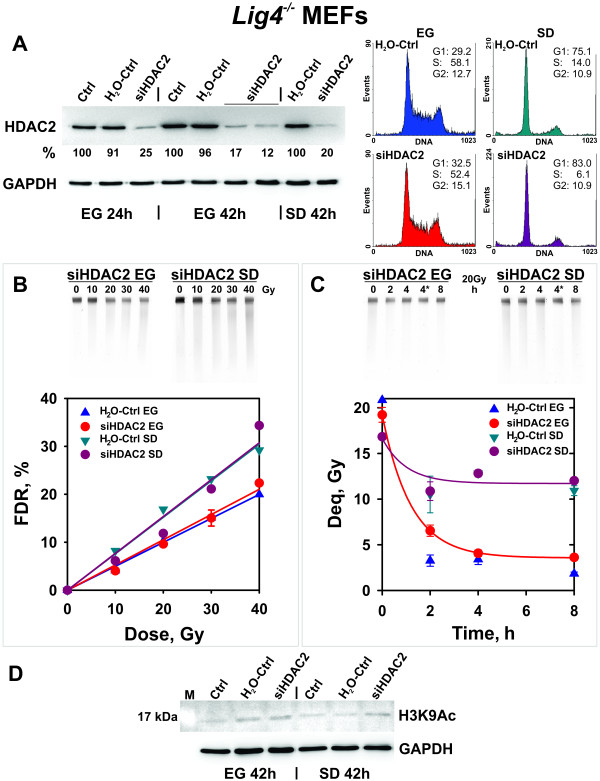
**Effect of *HDAC2 *silencing on B-NHEJ in serum deprived (SD) versus exponentially growing (EG) *Lig4***^***-/- ***^**MEFs. (A)** Western blot of EG and SD cells at different times after transfection of siRNA targeting *HDAC2* together with the corresponding controls. The right panel depicts the cell cycle distribution of cells used in the radiation experiments shown in the following panels. Other details are as in Figure 1A. **(B)** and **(C)** Induction and repair of DSBs. Other details are as in 1B and 1C. The results shown represent the mean and standard error from 2 - 3 determinations in one experiment. Irradiation and DSB repair analysis was performed 40 h after transfection. 4* represents a non-irradiated control measured at 4 h. Serum deprived cells were prepared and treated as described under “Methods”. **(D)** Histone H3 acetylation (H3K9Ac) in control and *HDAC2* silenced MEFs together with the corresponding controls.

We considered the possibility that the reduction in B-NHEJ observed in cells that enter the plateau-phase of growth, or in serum-deprived cells, is mediated by some form of chromatin condensation. Therefore, we examined whether the expected chromatin decondensation following *HDAC2* knockdown modulates B-NHEJ in plateau-phase *Lig4*^-/-^ MEFs. In these experiments, cells were transfected with *HDAC2* siRNA and grown in complete medium for 24 h. They were subsequently transferred to serum-free medium and irradiated 16 h later. At this time a strong accumulation of cells in G1 is observed for the serum-deprived (SD) as compared to the exponentially growing (EG) samples. Figure 2A shows that this protocol achieves efficient knockdown for *HDAC2* not only in exponentially growing, but also in the serum-deprived cells. The same figure also demonstrates that the cell cycle distribution of cells exposed to siRNA is as expected from the growth conditions applied and is not detectably affected by the *HDAC2* knockdown. The same holds true for the dose–response curves for DSB induction by IR generated with the different cell populations (Figure 2B). Here again the expected increase in FDR is observed in cells entering G1 as a result of serum deprivation [58], but *HDAC2* knockdown has no additional effect. As expected, serum deprivation compromises B-NHEJ (Figure 2C). However, this reduction in B-NHEJ efficiency cannot be reversed by *HDAC2* knockdown, despite the efficient protein down regulation achieved (Figure 2A). 

We inquired whether the efficient silencing of *HDAC2* modifies the acetylation status of chromatin in *Lig4*^-/-^ MEFs. Figure 2D shows that despite nearly 90% depletion of HDAC2, chromatin acetylation remains low both in exponentially growing as well as in serum-deprived cells. We conclude that multiple HDACs contribute to histone deacetylation in *Lig4*^*-/-*^ MEFs, and that as a consequence inhibition of HDAC2 alone fails to generate detectable effects on chromatin acetylation.

### Effect of TSA on chromatin acetylation and B-NHEJ

**Figure 3:**
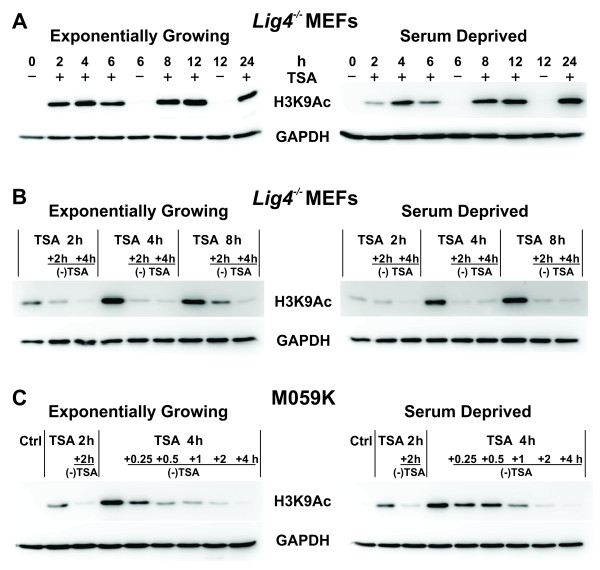
**Histone H3 acetylation and deacetylation in TSA-treated *Lig4***^***-/- ***^**MEFs analyzed either during exponential growth (EG) or after serum deprivation (SD).(A)** Western blot analysis for histone H3 hyperacetylation (H3K9Ac) after treatment for different times of EG or SD cells with 0.5 μM TSA. Controls (Ctrl) were incubated with DMSO. **(B)** Time dependent loss of histone H3 acetylation in EG and SD *Lig4*^*-/-*^ MEFs after incubation with TSA for the indicated periods of time. Other details are as in 3A. **(C)** As in panel B but for M059K cells.

The lack of histone hyperacetylation following *HDAC2* knockdown suggested that inhibition of multiple HDACs is required for global changes in chromatin acetylation. Therefore, we tested TSA, a non-specific inhibitor of class I and II HDACs (see Introduction). Treatment of *Lig4*^-/-^ MEFs with 0.5 μM TSA causes strong hyperacetylation of H3K9Ac, detectable already 2 h after drug administration that is maintained for up to 24 h. This effect is observed both in exponentially growing, as well as in serum-deprived cells, although hyperacetylation occurs faster in growing cells (Figure 3A).

TSA mediated H3K9Ac is reversible within about 2 h of drug removal, irrespectively of treatment duration between 2-8 h, in exponentially growing and serum deprived *Lig4*^-/-^ MEFs (Figure 3B), as well as in the human tumor cell line, M059K (Figure 3C).

**Figure 4:**
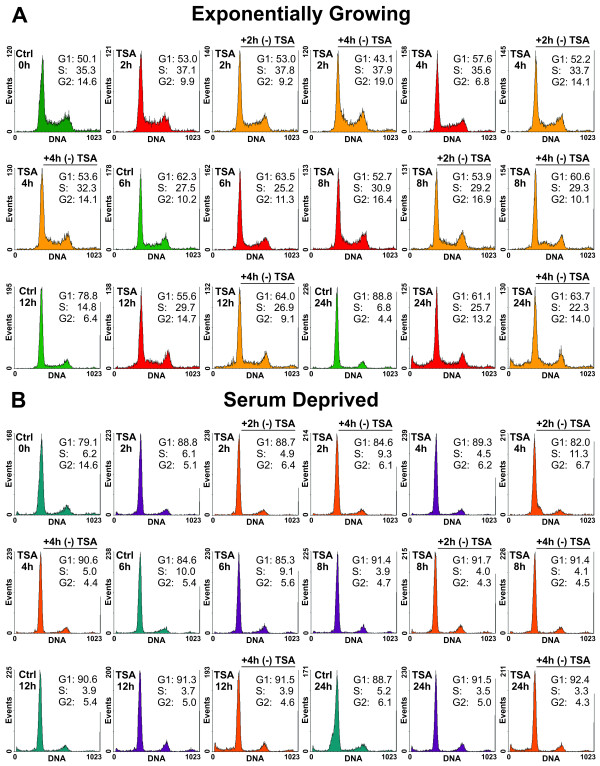
**Cell cycle distribution of TSA treated *Lig4***^***-/- ***^**MEFs.** Cell cycle distribution of exponentially growing (EG) and serum deprived (SD) *Lig4*^*-/-*^ MEFs after treatment for different periods of time with 0.5 μM TSA. Shown are also results obtained with cells analyzed at different times after completion of TSA treatment. **(A)** Results obtained with EG cells. **(B)** Results obtained with SD cells.

The cell cycle distribution of TSA-treated exponentially growing *Lig4*^-/-^ MEFs shows accumulation in S-phase and the formation of a sub-G1 peak indicative of apoptotic cell death after prolonged (12 - 24 h) incubation with the drug (Figure 4A). Cell cycle effects and toxicity, evidenced as sub-G1 peak, are not detectable in serum deprived cells (Figure 4B). We conclude that TSA causes fast and reversible global changes in chromatin acetylation, irrespectively of growth-state, within 2-4 h without overly affecting cellular integrity or the distribution of cells throughout the cell cycle.

**Figure 5:**
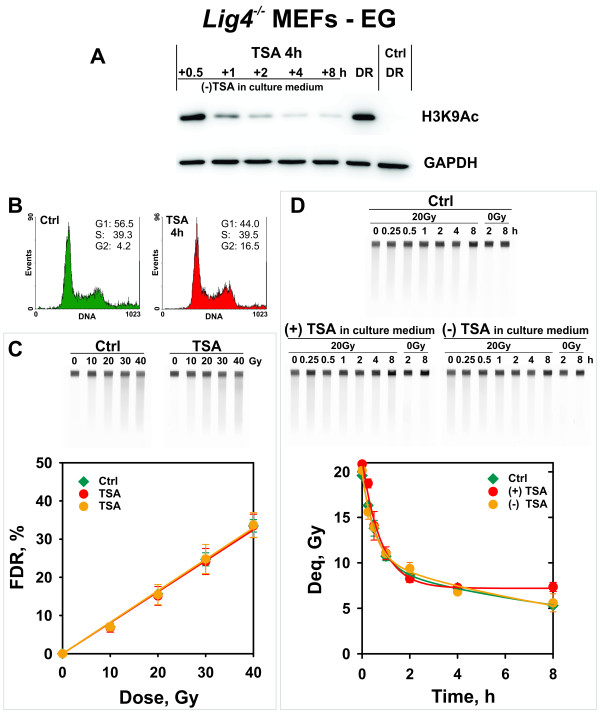
**B-NHEJ kinetics in TSA-treated exponentially growing *Lig4***^***-/- ***^**MEFs. (A)** Western blot showing H3K9Ac acetylation at the time of IR exposure 4 h after TSA administration, as well as at different times after drug removal. DR - cells used for the dose response curve; correspond to acetylation measured after 4 h TSA treatment; Ctrl – cells treated with DMSO. **(B)** Cell cycle distribution of control and TSA-treated cells employed in DSB repair experiments. Cells were analyzed immediately before exposure to IR. **(C)** Induction of DSBs in cells treated as indicated. Other details are as in Figure 1B. **(D)** Kinetics of rejoining of IR induced DSBs in control and TSA-treated cells incubated for repair in the presence (+) or absence (-) of TSA. The results shown represent the mean and standard error calculated from 6 determinations in 2 independent experiments. Other details are as in Figure 1C.

To study the effect of global chromatin hyperacetylation on B-NHEJ, exponentially growing *Lig4*^-/-^ MEFs were treated with 0.5 μM TSA for 4 h and subsequently exposed to 20 Gy X-rays. After IR, one set of dishes was incubated with TSA for repair, whereas a second group of dishes was transferred to TSA-free growth medium for repair. Figure 5A shows the level of hyperacetylation achieved and the kinetics of loss of this hyperacetylation upon TSA removal. There are only minor changes observed in cell cycle distribution in cells treated with TSA (Figure 5B). Despite the strong hyperacetylation observed and the presumed chromatin decondensation, induction of DSBs by IR remains unchanged (Figure 5C). Notably, extensive chromatin hyperacetylation leaves unchanged the kinetics of DSB rejoining by B-NHEJ (Figure 5D).

**Figure 6:**
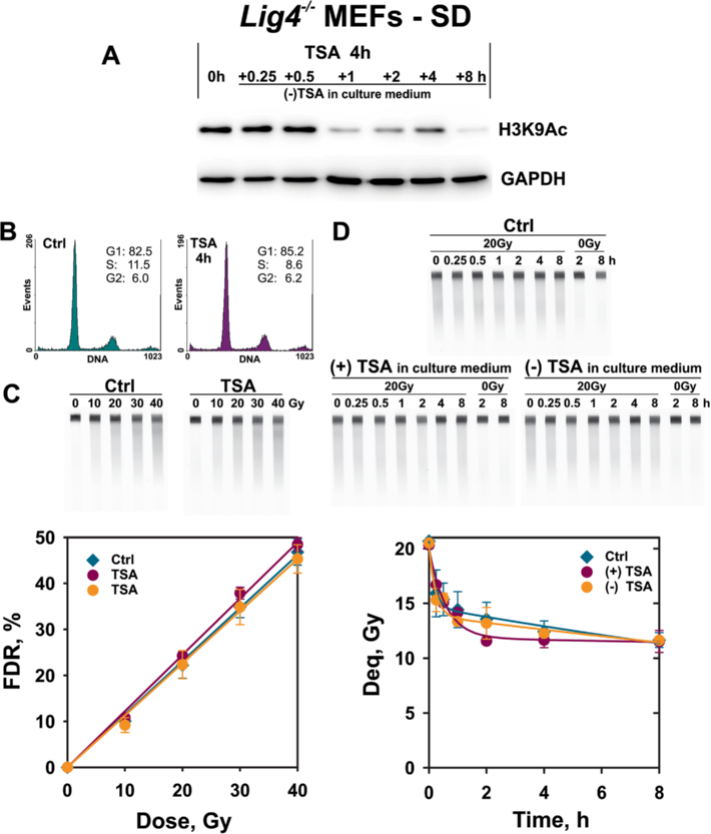
**B-NHEJ kinetics in TSA treated serum deprived (SD) *Lig4***^**-/- **^**MEFs.** As shown in Figure 5, but for cells treated with TSA after serum deprivation. Panel D shows the mean and standard error of 8 determinations in 2 independent experiments.

TSA treatment as described above but for serum-deprived *Lig4*^-/-^ MEFs (Figure 6) shows a prolonged persistence of hyperacetylated chromatin (Figure 6A) without significant shifts in cell cycle distribution (Figure 6B). Here again, the dose response curves for DSB induction are not affected by histone hyperacetylation (Figure 6C) and repair of DSBs, although overall reduced in the untreated controls, remains unchanged in TSA-treated samples (Figure 6D).

To rule out species-specific differences in the response to hyperacetylation, we carried out experiments using a human cell system. HCT116 *Lig4*^-/-^ cells, either exponentially growing or after serum deprivation, were treated with TSA under conditions similar to those described above for *Lig4*^-/-^ MEFs and cell cycle distribution, as well as induction and repair of DSBs were measured. The results summarized in Additional files: 2 and 3 show that histone hyperacetylation has only a small effect on the yields of DSBs, as well as the kinetics of their repair.

**Figure 7:**
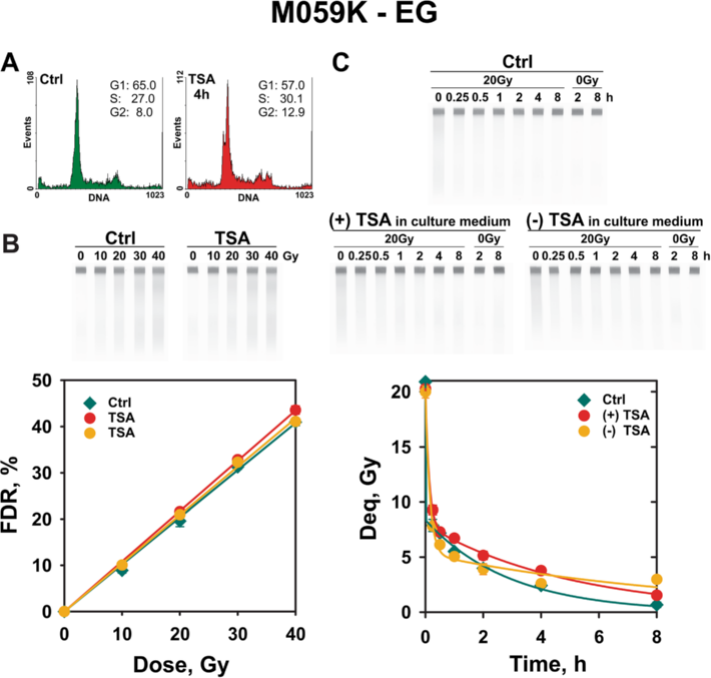
**B-NHEJ kinetics in TSA-treated exponentially growing M059K cells incubated for repair in the presence of 20 μM wortmannin.** Other details are as shown in Figure 5 for *Lig4*^*-/- *^ MEFs. M059K cells were subjected to 20 μM wortmannin treatment 40 min before the beginning of the experiment in order to inhibit D-NHEJ and allow thus analysis of B-NHEJ activity. Data shown are the means and standard errors of four determinations in one experiment.

**Figure 8:**
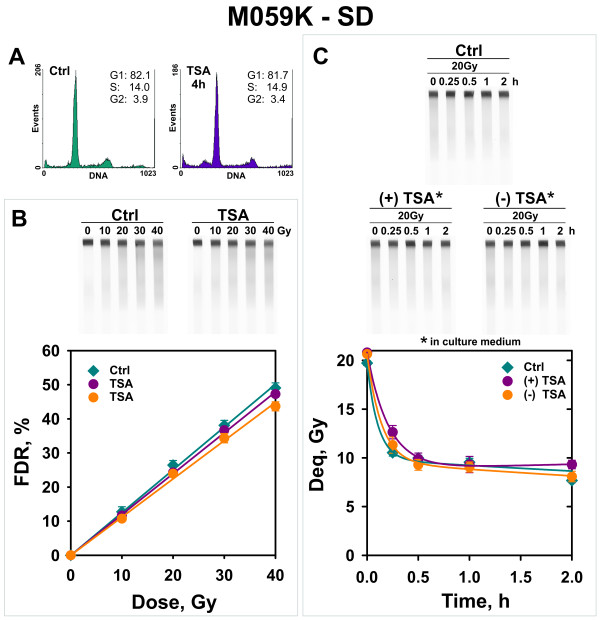
**B-NHEJ kinetics in TSA-treated serum deprived M059K cells incubated for repair in the presence of 20 μM wortmannin.** Other details are as shown in Figure 6 for SD *Lig4*^*-/- *^ MEFs. M059K cells were subjected to 20 μM wortmannin treatment 40 min before the beginning of the experiment in order to inhibit D-NHEJ and allow thus analysis of B-NHEJ activity. Data shown are the means and standard errors of four determinations in one experiment.

Lastly, we investigated the effect on DSB repair of histone hyperacetylation in the D-NHEJ proficient M059K cells. In these cells, we use wortmannin at 20 μM to inhibit D-NHEJ and study effects on B-NHEJ. Here again, experiments were carried out with exponentially growing and serum-deprived cells. The results summarized in Figures 7 and 8 clearly show that chromatin hyperacetylation leaves unchanged the yield of DSBs as a function of radiation dose, as well as the kinetics of B-NHEJ. In this set of experiments, serum deprived cells maintained in TSA for repair displayed strong wortmannin toxicity after 2 h of incubation. Therefore analysis was restricted to 2 h postirradiation.

## Discussion

The present study was designed with the purpose of analyzing the effect of chromatin structure as determined by histone acetylation on the efficiency of B-NHEJ. Particular emphasis was placed on investigating whether alterations in chromatin structure underpin the reduced function of B-NHEJ observed in non-cycling cells.

Since HDAC inhibitors typically target multiple histone deacetylases, which complicates the assignment of an effect to a specific enzyme [59], we began our experiments using RNA interference that allows the suppression of specific HDACs. As target we selected *HDAC2* which has been implicated in DNA damage response [33]. Our results show that *HDAC2* downregulation leaves unchanged the yield of DSBs after IR in both exponentially growing and serum deprived *Lig4*^*-/-*^MEFs, despite its documented role in the regulation of chromatin plasticity and structure [60]. Also the ability of cells to remove DSBs by B-NHEJ remains unaffected after *HDAC2* knockdown and this response is observed again both in actively growing, as well as in serum deprived cells (Figures 1 and 2).

While this result suggests that B-NHEJ remains unaffected by changes in chromatin conformation, it is also possible that *HDAC2* suppression fails to relax chromatin to levels sufficient to modulate the efficiency of B-NHEJ. In addition, our data show a negligible effect of HDAC2 depletion on the level of histone acetylation, which may preclude modulation of B-NHEJ.

Recent reports suggest that changes in chromatin acetylation and modulation of DSB repair require knockdown of multiple HDACs [40,61]. To address this possibility and inhibit multiple HDACs, we introduced the non-specific HDAC inhibitor TSA. TSA causes histone hyperacetylation, modulates the transcription of certain groups of genes and alters cell cycle progression [62]. In *Lig4*^-/-^ MEFs, treatment with TSA for 4 h causes marked hyperacetylation both in actively growing, as well as in serum deprived cells with no signs of toxicity (Figures 3 and 4). On the other hand, prolonged incubation with TSA causes cell death, possibly by apoptosis, as already reported for other cell systems [63,64]. Notably, TSA-induced chromatin hyperacetylation is for the most part reversible within 2-4 h after drug removal (Figure 5).

Despite its strong histone hyperacetylation potential, TSA fails to modulate B-NHEJ in actively growing cells where it functions robustly and removes nearly 80% of the induced DSBs within 8 h (Figure 5). TSA fails to modulate DSB repair in serum deprived cells as well, where B-NHEJ is markedly compromised as compared to actively growing cells [4,27,65]. Even prolonged incubations with TSA fail to modulate B-NHEJ under these conditions (results not shown).

We had hypothesized that the reduced function of B-NHEJ in serum deprived cells partly derives from chromatin compaction associated with the transition of cells to a quasi-Go state and speculated that chromatin relaxation after treatment with TSA will rescue B-NHEJ activity. The results obtained clearly demonstrate that this is not the case and suggest that B-NHEJ remains rather immune to changes in chromatin conformation. This likely reflects its backup nature, which requires B-NHEJ to remain functional in a wide variety of conditions, including different states of chromatin compaction, albeit at the price of a lower overall efficiency.

HDAC inhibitors in general and TSA in particular, modulate cell cycle progression by inducing G1/S and/or G2/M arrest in both normal and tumor cells [62]. Our flow cytometry results show a slight accumulation of cells in G2 (Figure 4), in agreement with observations in HeLa cells [48]. Since B-NHEJ is known to have a marked cell cycle component, redistribution of actively growing cells throughout the cell cycle after treatment with TSA may mask small modulations in B-NHEJ activity. However, since changes in cell cycle distribution are not observed in actively growing cells earlier than 12 h after treatment begin, such effects seem unlikely. This is also in line with the observation that serum deprived cells, which are immune to treatment-related cell cycle fluctuations, fail to show modulations in B-NHEJ efficiency after treatment with TSA. Therefore, we conclude that histone H3 hyperacetylation does not affect B-NHEJ under the experimental conditions tested.

Notably, similar results are obtained with the human HCT116 *Lig4*^-/-^ mutant, as well as with the D-NHEJ proficient M059K cells, in which B-NHEJ activity is tested by inhibiting D-NHEJ via treatment with the DNA-PKcs inhibitor wortmannin [28]. In summary, our combined results lead us to propose that parameters beyond chromatin acetylation or deacetylation determine B-NHEJ efficiency in the plateau-phase of growth in all types of cells examined.

It is worth noting that treatment with TSA and the associated histone hyperacetylation and chromatin relaxation leave unchanged the yield by IR of DSBs. In HeLa cells, significant chromatin decondensation occurs 4 h after TSA treatment, and condensed chromatin reappears hours after TSA removal [48]. We therefore infer that in our experimental systems similar changes in chromatin conformation also occur, but that these changes may not be of sufficient magnitude to affect the yield of radiation-induced DSBs. Alternatively, it is possible that changes in chromatin conformation only affect subsets of DSBs that cause fragmentation of chromatin [66]. Further work will be required to address this important question.

While the above experiments do not establish links between B-NHEJ function and chromatin structure, arguments can be developed as to why chromatin structure may affect end joining. Thus, induction of a DSB in a condensed region of chromatin is likely to limit the diffusion of ends and facilitate their rejoining. Linker histones may be particularly helpful in this regard [12]. Specifically for B-NHEJ, which is inherently slow, relaxed chromatin might increase the chance of end synapsis - even with the wrong ends. Only further work will elucidate the complex contributions of chromatin structure on DSB repair in general [67] and the function of B-NHEJ in particular. The present study is a first step in this direction.

## Conclusions

Cell cycle and growth-state regulation of B-NHEJ differ fundamentally from that of other DSB repair pathways. B-NHEJ shows a marked enhancement during the G2 phase and is markedly compromised in cells that enter the plateau-phase of growth, thus suggesting the involvement of important regulatory mechanisms that remain to be elucidated. The present work examines chromatin conformation as a possible modulator of B-NHEJ efficiency. Our results show that histone H3 hyperacetylation induced by short-term TSA treatment does not affect the operation of B-NHEJ under the experimental conditions employed. We propose that parameters beyond chromatin acetylation or deacetylation determine the reduced efficiency of B-NHEJ in the plateau-phase of growth and that B-NHEJ may be flexible enough to operate efficiently in a wide variety of chromatin states – true to its backup nature.

## Methods

### Cells and culture conditions

*Lig4*^-/-^ MEFs (a gift of Dr. Frederick W. Alt, Harvard Medical School, Boston, MA) [68] and their wild-type counterparts were maintained in Dulbecco`s Modified Eagle`s Medium (D-MEM, Sigma-Aldrich) supplemented with 10% fetal bovine serum (FBS) and antibiotics. M059K cells (a gift of Dr. Joan Allalunis-Turner, University of Alberta, Edmonton, AB) [69] were also grown in D-MEM supplemented with 10% FBS. HCT116 *Lig4*^*-/-*^ cells (a gift of Dr. Eric A. Hendrickson, University of Minnesota, Minneapolis, MN) [70] were grown in McCoy’s 5a medium supplemented with 10% FBS and antibiotics. Experiments were carried out with both exponentially growing (EG), as well as with cells that were transferred for 24 h to serum-free medium. Serum-deprivation (SD) causes cells to stop growing and to enter a plateau-phase [29]. To generate serum-deprived cultures, cells were seeded as usual and were allowed to grow for 24 h. Subsequently cultures were transferred to serum-free medium and were used for experiments 16-48 h later depending on cell type. With this protocol, serum-deprived cultures with more than 80% cells in G1 phase could be generated.

Cell cycle distribution was routinely monitored by flow cytometry. For this purpose, cells were fixed in ethanol and stained with propidium iodide as previously described [25]. Samples were analyzed in a Beckman Coulter flow cytometer (Excel-MCL).

### *HDAC2* knockdown

To knockdown *HDAC2* in *Lig4*^*-/-*^ MEFs we tested four small interfering RNAs (siRNAs) targeted against different domains of the mouse *HDAC2* transcript (Qiagen). AllStars siRNAs (Qiagen) were used as a negative control. Actively growing cells (5×10^6^) were transfected with 2500 ng siRNAs by electroporation using the MEF1 kit and the T-20 program of the Nucleofector device (Amaxa, Germany). Three controls were run in parallel: 1. Negative control; cells electroporated with 2500 ng non-silencing siRNAs (siNc). 2. Mock-transfection control; cells subject to electroporation in MEF1 solution using water instead of siRNA (H_2_O-Ctrl). 3. Non-treated control; cells not subject to transfection solution and not electroporated (Ctrl).

After transfection, cells were plated in 60 mm tissue culture dishes in 5 ml pre-warmed growth medium and returned to normal incubation conditions; non-electroporated cells were seeded at 0.2×10^6^/dish and electroporated cells at 0.4×10^6^ to account for 50% cell loss due to the electroporation shock. After cell attachment culture medium was replaced to remove debris and dead cells. The level of knockdown was monitored by western blotting and real-time RT-PCR.

### Treatment with TSA

Histone hyperacetylation was provoked by treatment of cells with 0.5 μM TSA (dissolved in DMSO) for different incubation time intervals ranging from 2 to 24 h. Drug was added to cells 4 h before IR and control cells were treated with DMSO only. To follow B-NHEJ kinetics of TSA-treated cells in the absence of TSA, culture medium was replaced with TSA-free medium immediately after IR.

### Western blotting

Cells were trypsinized, counted and an equal number collected by centrifugation. Pellets (0.5×10^6^ cells) were resuspended in SDS sample buffer (100 μl) and sonicated in an ultrasonic water bath at 75°C. Whole-cell extracts of 0.25 or 0.5×10^5^ cells were run in a 10% SDS-PAGE gel and transferred to a PVDF membrane. As primary antibody against HDAC2 the Mab-HDAC2 monoclonal antibody (Abcam) was used, at a 1:2000 dilution; as secondary antibody an HRP-linked anti rabbit IgG (Cell Signaling, 1:1000) was used. GAPDH protein, detected by the primary antibody GAPDH (Chemicon, Int., 1:50 000 dilution) was used as a loading control.

TSA-induced chromatin hyperacetylation was assessed by monitoring acetylation of histone H3 at Lys9 (H3K9Ac). Cells were collected, washed with PBS and frozen at -20°C. Pellets were processed as described above and whole-cell extracts of 0.5×10^5^ cells were electrophoretically separated in 12.5% SDS-PAGE gels before transferring to PVDF membranes. As primary antibody against H3K9Ac the monoclonal Ab4441 (Abcam) was used, at 1:2000 dilution; secondary antibody was the HRP-linked anti rabbit IgG (Cell Signaling, 1:2000).

Proteins were visualized using the ECL-Plus kit (GE Healthcare) and signal was captured with the VersaDoc (Bio-Rad). Densitometry analysis of digitalized images was performed by ImageQuant TL 7.0 (GE Healthcare) software.

### Quantitative real-time RT-PCR

RNA was extracted using the "High Pure RNA" isolation kit (Roche) and RNA concentration determined in the Nanodrop (Thermo). Total RNA (500 ng/reaction) was reverse-transcribed using "Transcriptor First Strand" cDNA synthesis kit (Roche). Real time PCR analysis was performed with "LightCycler-DNA Master SYBR Green I" reaction mix (Roche) in LightCycler 2.0 (Roche, Mannheim, Germany). The primers for amplifying a 64 bp fragment of mouse *HDAC2* were from Qiagen (QuantiTect primer assay). The mouse *Tbp* gene was used as reference. The percentage of mRNA reduction is estimated based on the relative expression ratio of *HDAC2* gene calculated according to Pfaffl, 2001 [71] (efficiency of amplification and ΔCp of *HDAC2* gene in siHDAC2 treated sample and its control, normalized to *Tbp* gene as a reference).

### Treatment with Wortmannin

To follow B-NHEJ in M059K cells after treatment with TSA, D-NHEJ was inhibited using the irreversible DNA-PKcs inhibitor wortmannin. The drug (20 μM) was added 40 min before IR.

### Pulsed-field gel electrophoresis

Repair of DSBs was analyzed by pulsed-field gel electrophoresis (PFGE) as previously described [72]. Exponentially growing cells were cooled for 15 min and irradiated on ice; serum-deprived cells were irradiated at room temperature. Irradiations were carried out with an X-ray machine (GE Healthcare, 320 kV, 12 mA) at a dose rate of 2.7 Gy/min and a distance of 50 cm. After irradiation cells were incubated for repair in pre-warmed fresh growth medium for the indicated periods of time. Subsequently, they were trypsinized, collected on ice and embedded in low-melting-point agarose. The resulting agarose blocks were incubated at 50°C for 18 h in a lysis solution containing 0.2 mg/ml protease A.

After completion of lysis and extensive washing, agarose blocks were loaded on 0.5% agarose gels and subjected to asymmetric field inversion gel electrophoresis (AFIGE) (cycles of 1.25 V/cm for 900 s in the direction of DNA migration and 5 V/cm for 75 s in the reverse direction) in 0.5×TBE at 10°C for 40 h. Gels were subsequently scanned in the "Typhoon" (GE Healthcare) and analyzed using ImageQuant 5.2 (GE Healthcare). The fraction of DNA released (FDR) from the well into the lane was used as a measure of DSBs present in the cells. For a quantitative analysis of DSB repair kinetics, the equivalent dose (Deq) was determined for each FDR value from a dose–response curve generated in parallel using the same cell population. To generate these dose–response curves, cells were first embedded in agarose and then irradiated on ice in serum-free medium with increasing X-ray doses. Immediately after irradiation agarose blocks were processed for lysis and PFGE as described above.

## Competing interests

The authors declare that they have no competing interests.

## Authors’ contributions

VM, SKS and GI designed the experiments; VM performed knock-down, real-time RT-PCR and Western blot experiments; VM and SKS performed TSA treatment and PFGE. VM, SKS and GI analyzed the data; VM and GI wrote the manuscript. All authors read and approved the final manuscript.

## Supplementary Material

Additional file 1 *** HDAC2 *knock-down in *Lig4***^***-/- ***^**MEFs.** (A) Western blot analysis showing depletion of the target protein in siHDAC2-transfected *Lig4*^*-/-*^ MEFs. Other details are as in Figure 1A. (B) Relative knockdown of HDAC2 mRNA in control and siHDAC2 transfected *Lig4*^*-/-*^ MEFs as determined by real-time RT-PCR. (C) Cell cycle distribution of *Lig4*^*-/-*^ MEFs treated with siRNA targeting *HDAC2,* together with the corresponding controls. Click here for file

Additional file 2 ** B-NHEJ in TSA-treated exponentially growing (EG) human colon tumor HCT116 *Lig4***^***-/- ***^**cells.** (A) Cell cycle distribution of control and TSA-treated cells employed in DSB repair experiments. Cells were analyzed immediately before exposure to IR. (B) Induction of DSBs in cells treated as indicated. (C) Kinetics of rejoining of IR induced DSBs in control and TSA-treated cells incubated for repair in the presence (+) or absence (-) of TSA. Data shown are the means and standard errors of four determinations in one experiment. Click here for file

Additional file 3 ** B-NHEJ in TSA-treated serum deprived (SD) human colon tumor HCT116 *Lig4***^***-/- ***^**cells.** (A) Cell cycle distribution of control and TSA-treated cells employed in DSB repair experiments. Cells were analyzed immediately before exposure to IR. (B) Induction of DSBs in cells treated as indicated. (C) Kinetics of rejoining of IR induced DSBs in control and TSA-treated cells incubated for repair in the presence (+) or absence (-) of TSA. Data shown are the means and standard errors of two determinations in one experiment. Click here for file
