# Supplementary figures and images for: Processing of DNA double strand breaks by alternative non-homologous end-joining in hyperacetylated chromatin
Source: Genome Integr. 2012 Aug 22;3:4. doi: 10.1186/2041-9414-3-4 (PMC3471266; doi:10.1186/2041-9414-3-4)

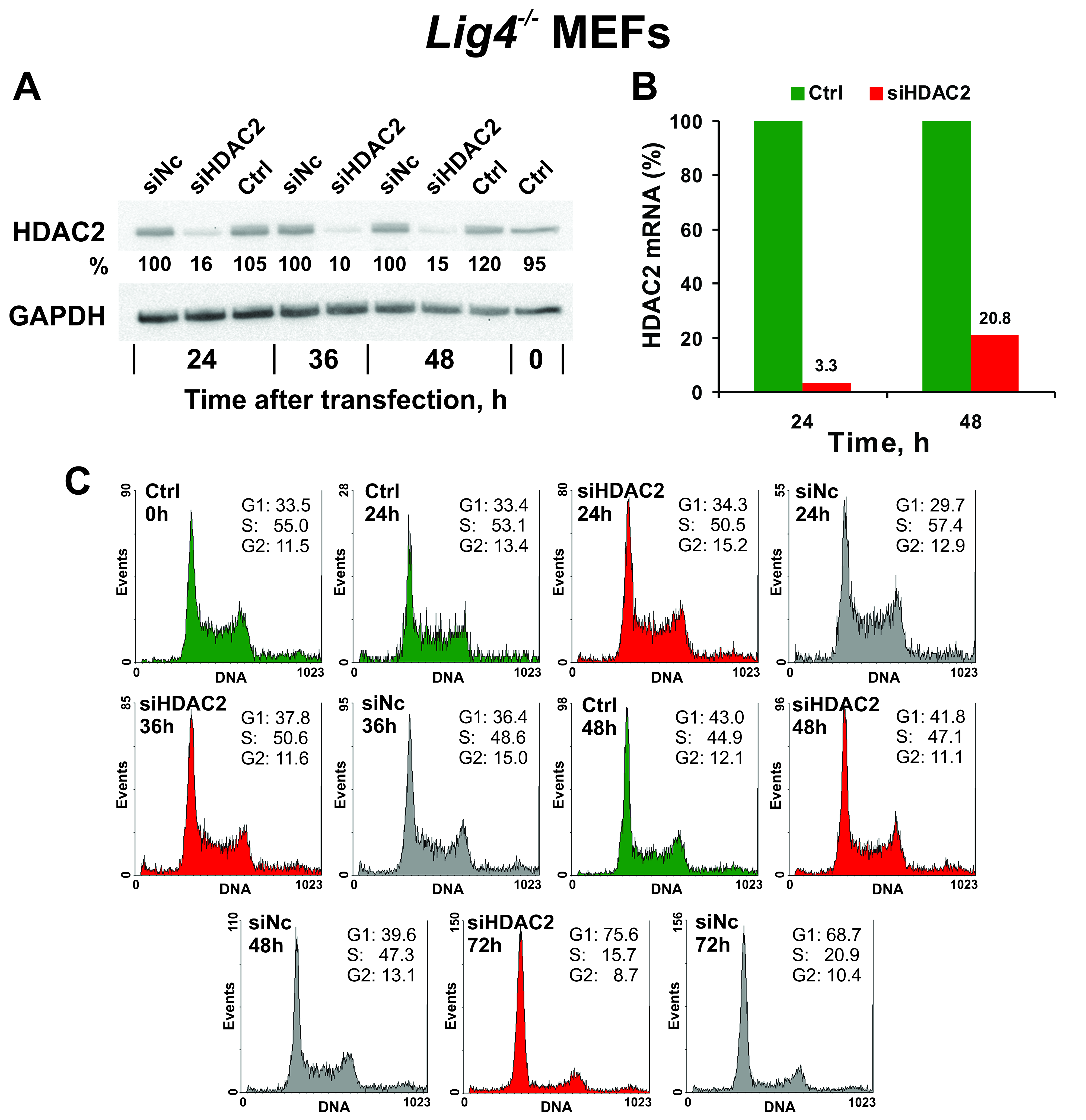

Supplement: Additional file 1 — HDAC2 knock-down in Lig4-/- MEFs. (A) Western blot analysis showing depletion of the target protein in siHDAC2-transfected Lig4-/- MEFs. Other details are as in Figure 1A. (B) Relative knockdown of HDAC2 mRNA in control and siHDAC2 transfected Lig4-/- MEFs as determined by real-time RT-PCR. (C) Cell cycle distribution of Lig4-/- MEFs treated with siRNA targeting HDAC2, together with the corresponding controls. [file 2041-9414-3-4-S1.tiff]

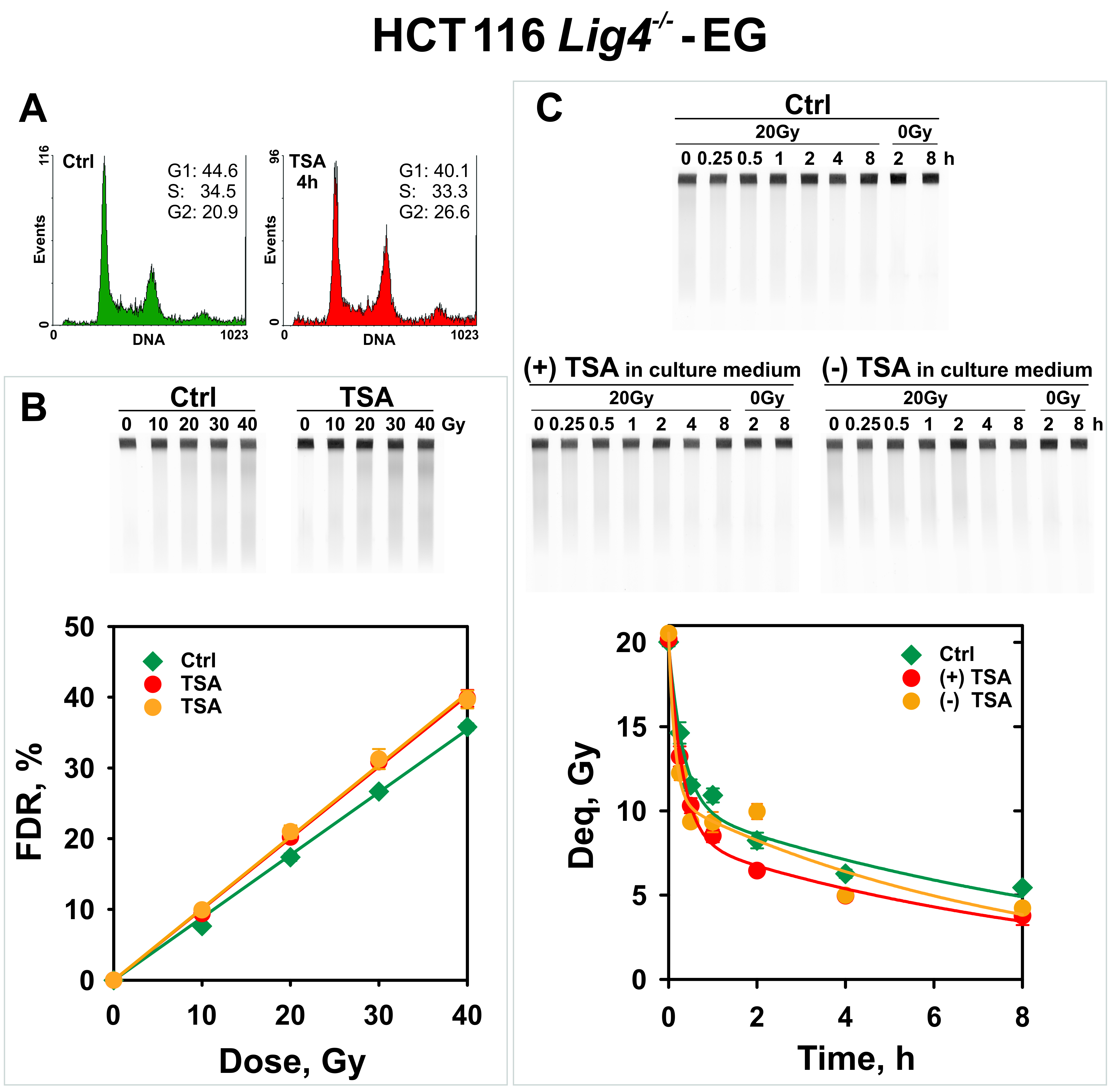

Supplement: Additional file 2 — B-NHEJ in TSA-treated exponentially growing (EG) human colon tumor HCT116 Lig4-/- cells. (A) Cell cycle distribution of control and TSA-treated cells employed in DSB repair experiments. Cells were analyzed immediately before exposure to IR. (B) Induction of DSBs in cells treated as indicated. (C) Kinetics of rejoining of IR induced DSBs in control and TSA-treated cells incubated for repair in the presence (+) or absence (-) of TSA. Data shown are the means and standard errors of four determinations in one experiment. [file 2041-9414-3-4-S2.tiff]

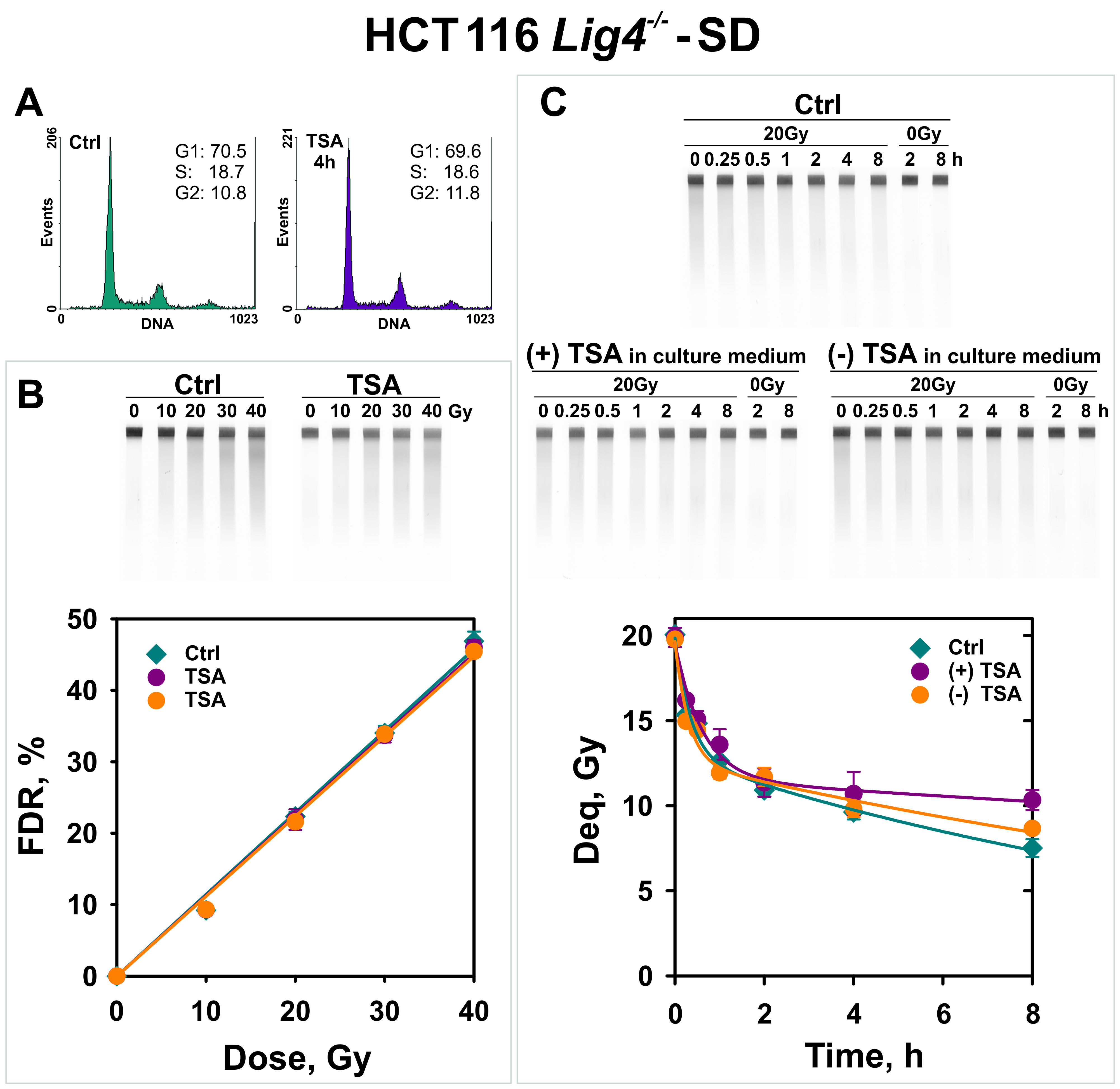

Supplement: Additional file 3 — B-NHEJ in TSA-treated serum deprived (SD) human colon tumor HCT116 Lig4-/- cells. (A) Cell cycle distribution of control and TSA-treated cells employed in DSB repair experiments. Cells were analyzed immediately before exposure to IR. (B) Induction of DSBs in cells treated as indicated. (C) Kinetics of rejoining of IR induced DSBs in control and TSA-treated cells incubated for repair in the presence (+) or absence (-) of TSA. Data shown are the means and standard errors of two determinations in one experiment. [file 2041-9414-3-4-S3.tiff]
